# Detection and characterization of clustered regularly interspaced short palindromic repeat-associated endoribonuclease gene variants in *Vibrio parahaemolyticus* isolated from seafoods and environment

**DOI:** 10.14202/vetworld.2019.689-695

**Published:** 2019-05-21

**Authors:** Pallavi Baliga, Malathi Shekar, Moleyur Nagarajappa Venugopal

**Affiliations:** Department of Fisheries Microbiology, Karnataka Veterinary, Animal and Fisheries Sciences University, College of Fisheries, Mangalore, Karnataka, India

**Keywords:** *cas6* gene, clustered regularly interspaced short palindromic repeats-cas operon, endoribonuclease, type IF system, *Vibrio parahaemolyticus*

## Abstract

**Aim:**

In *Vibrio parahaemolyticus*, the clustered regularly interspaced short palindromic repeat (CRISPR)-associated *cas6* endoribonuclease gene has been shown to exhibit sequence diversity and has been subtyped into four major types based on its length and composition. In this study, we aimed to detect and characterize the *cas6* gene variants prevalent among *V. parahaemolyticus* strains isolated from seafoods and environment.

**Materials and Methods:**

Novel primers were designed for each of the *cas6* subtypes to validate their identification in *V. parahaemolyticus* by polymerase chain reaction (PCR). In total, 38 *V. parahaemolyticus* strains isolated from seafoods and environment were screened for the presence of *cas6* gene. Few representative PCR products were sequenced, and their phylogenetic relationship was established to available *cas6* gene sequences in GenBank database.

**Results:**

Of the 38 *V. parahaemolyticus* isolates screened, only about 40% of strains harbored the *cas6* endoribonuclease gene, among which 31.6% and 7.9% of the isolates were positive for the presence of the *cas*6-a and *cas*6-d subtypes of the gene, respectively. The subtypes *cas6*-b and *cas6*-c were absent in strains studied. Sequence and phylogenetic analysis also established the cas6 sequences in this study to match GenBank sequences for *cas6*-a and *cas6-*d subtypes.

**Conclusion:**

In *V. parahaemolyticus*, the Cas6 endoribonuclease is an associated protein of the CRISPR-cas system. CRISPR-positive strains exhibited genotypic variation for this gene. Primers designed in this study would aid in identifying the *cas6* genotype and understanding the role of these genotypes in the CRISPR-cas immune system of the pathogen.

## Introduction

*Vibrio parahaemolyticus* is a Gram-negative, halophilic bacterium that naturally inhabits the marine, estuarine, and coastal environments. *V. parahaemolyticus* is commonly responsible for acute gastroenteritis illness and to lesser frequency wound infections and septicemia in humans [1]. Infections due to this bacterium are associated with the consumption of contaminated seafood [2,3] and exposure of open wounds to seawater [4]. Recently, *V. parahaemolyticus* has also been reported to be the causative agent of acute hepatopancreatic necrosis syndrome in penaeid shrimp taking a heavy toll on the shrimp aquaculture industry [5]. *V. parahaemolyticus* pathogenicity has been attributed to several virulence determinants including the well-known thermostable direct hemolysin (TDH) and TDH-related hemolysin encoded by the *tdh* and *trh* genes, respectively [6]. A recent study has demonstrated a significant association between the virulence factor *tdh* and the presence of clustered regularly interspaced short palindromic repeats (CRISPR)/cas in *V. parahaemolyticus* [7].

The CRISPR-cas (CRISPR-associated proteins) system is a prokaryotic adaptive immune system against genomic invaders such as viruses and plasmids [8,9]. Due to its importance and widespread applications, it has been a subject for comprehensive research recently [10]. The CRISPR-cas system consists of short (~25-40 bp) direct DNA repeats separated by spacer sequences and associated variable *cas* genes [8]. The Cas proteins are a diverse group of proteins which are believed to function as nucleases, helicases, and RNA-binding proteins [11]. Defense against foreign genetic elements is achieved in three stages: Adaptation, expression, and interference. The adaptation stage involves the integration of foreign DNA fragments into the bacterial genome at the CRISPR loci. The second stage is characterized by the expression of *cas* genes and the transcription of the CRISPR loci to generate a crRNA precursor (pre-crRNA) which is later processed into mature-crRNAs. During interference, the target nucleic acid is recognized and destroyed by the combined action of crRNA and Cas proteins [11,12]. The CRISPR-cas system has been identified in several bacterial and archaeal genomes [13,14] and broadly classified into five major types (I-V) and 16 subtypes (IA-F, I-U, IIA-C, IIIA-D, IV, and V), based on the organization of the CRISPR locus, *cas* gene assembly, and their interference mechanisms [15]. Studies show that different types of CRISPR-cas systems have evolved distinct mechanisms for a mature crRNA generation [16]. In type I-III systems, the mature-crRNA is generated specifically by the Cas protein Cas6 [17]. The Cas6 is a member of the RNA-binding protein superfamily called repeat-associated mysterious proteins (RAMPs) [18] which play a central role in generating guide RNAs for invader defense in prokaryotes [19]. The Cas6 proteins are also reported to be highly divergent and are thought to have coevolved with the highly variable CRISPR RNA repeat sequences [13].

In our previous study, based on computational analysis of existing *V. parahaemolyticus* *cas6* gene sequences in database, we have shown the existence of four subtypes for *cas6*, designated *cas6*-a, *cas6*-b, *cas6*-c, and *cas6*-d [20]. In this study, we designed novel primers for the polymerase chain reaction (PCR) detection and characterization of *cas6* gene variants in *V. parahaemolyticus*. We report the variant types harbored for the Cas6 endoribonuclease in *V. parahaemolyticus*. Few of the *cas6* sequences were sequenced to look for further variations within the variant types.

## Materials and Methods

### Ethical approval

Ethical approval was not applicable, as this study does not involve any human or animal studies.

### Bacterial strains

*V. parahaemolyticus* stock cultures (n=38) maintained in Tryptic soy broth with 30% glycerol at −80°C at the Department of Fisheries Microbiology, College of Fisheries, Mangalore, were used in the experiments. The cultures were revived by growing them overnight in 5 ml of Luria-Bertani (LB) broth (HiMedia Laboratories Private Limited, Mumbai, India) at 37°C in a shaker incubator. A loopful of the culture was streaked onto thiosulfate-citrate-bile salts-sucrose agar (HiMedia Laboratories Pvt. Limited, Mumbai, India) to check for the purity of the cultures. *V. parahaemolyticus* strains used in this study are listed in Table-1.

**Table-1:** *Vibrio parahaemolyticus* isolates used in this study.

S.No	Isolate	Isolation region	Isolation source	Presence of *cas6*/subtype
1	VP 1	Karwar	Shrimp	+/d
2	VPh 2	Karwar	Shrimp	−
3	VPh 3	Karwar	Shrimp farm sediment	+/d
4	VP 15	Sasthan	Shrimp farm water	+/a
5	VPh 4	Karwar	Shrimp farm water	−
6	VP 5	Karwar	Shrimp farm sediment	+/d
7	VPh 6	Karwar	Shrimp farm water	−
8	VPh 8	Kundapur	Shrimp	−
9	VPh 9	Kundapur	Shrimp farm water	−
10	VP 4	Mulki	Clam	−
11	VPh 10	Kundapur	Shrimp farm water	−
12	VPh 11	Ankola	Shrimp	−
13	VPh 7	Kundapur	Shrimp	−
14	VPh 12	Ankola	Shrimp	−
15	VPh 13	Ankola	Fish	−
16	VP 6	Mulki	Oyster	+/a
17	MR 32	Sasthan	clams	+/a
18	MR 34	Sasthan	clams	−
19	125	Gangolli	Shrimp farm water	−
20	IIWVp	Katpadi	Shrimp farm water	−
21	VPh 1	Sasthan	Oysters	−
22	VP 3	Sasthan	Oyster	−
23	VP 9	Sasthan	Oyster	+/a
24	VP 10	Sasthan	Clam	+/a
25	VP 11	Sasthan	Oyster	−
26	VP 7	Mulki	Clam	+/a
27	VP 8	Sasthan	Oyster	+/a
28	VP 14	Mulki	Oyster	+/a
29	VP 18	Mulki	Fish	−
30	VP 19	Sasthan	Oyster	−
31	VP 20	Mulki	Oyster	−
32	VP 21	Mulki	Clam	−
33	VP 22	Sasthan	Clam	−
34	VP 23	Mulki	Oyster	+/a
35	VP 25	Mulki	Clam	+/a
36	VP 27	Sasthan	Clam	+/a
37	VP 17	Sasthan	Clam	+/a
38	VPh 5	Mulki	Oyster	−

### Primer design and PCR validation of *cas6* gene variants

The primers used in this study were designed based on respective *cas6* gene sequences available in GenBank database, using the Primer 3.0 software [21]. The primer sequences are listed in Table-2.

**Table-2:** Primers designed in this study for the detection of *cas6* gene variants.

Gene	Primer	Sequence (5’- 3’)	Annealing temperature	Expected product size
*cas6*-a (internal)	*cas6*-a F	CCAAGAAACGGTGGGACGTA	60°C	245 bp
	*cas6*-a R	CGCGTTCTAAAGCTCTTCGC		
*cas6*-a (full)	IF-a-F	GAACCATCACATTTTTACCTGAA	50°C	597 bp
	IF-a-R	CAATGGAACAACCTGCAATG		
*cas6*-b	IF-b-F	TTATTGGCGGGTCGCTGTAT	60°C	506 bp
	IF-b-R	TTCGTTGCGAGTCCGTAACT		
*cas6*-c	IF-c-F	GAAGCATTAATCGGGCACTG	51°C	500 bp
	IF-c-R	GTCCATAACTTGAGAATGCCC		
*cas6*-d	IF-d-F	TGGACTACTACCAAGAAATTAC	46°C	600 bp
	IF-d-R	TAAAATTGTGGGACAGTC		

### PCR and sequencing of *cas6* gene

Crude lysate of each strain was prepared by first growing them on LB broth at 37°C. 450 µl of ×1 TE buffer was added to 50 µl of the culture and subjected to heating at 94°C for 1 min in the hot dry bath followed by snap cooling in ice. PCR for the identification of *cas6* gene type was carried out using primers designed to target the *cas6* gene types. The PCR was carried out in a 30 µl reaction mixture consisting of 3 µl of ×10 PCR buffer (Genei^™^, Merck Bioscience, Bengaluru), 50 µM each of the four deoxynucleotide triphosphates, 10 pmol of each primer, and 1.0 U of *Taq* DNA polymerase (Genei^™^, Merck Bioscience, Bengaluru). 2 µl of crude lysate was used as DNA template. The PCR assay was carried out in a Programmable Thermocycler (PTC 200, Bio-Rad, CA) with the program as follows: Initial denaturation at 94°C for 5 min, 30 cycles of 94°C for 1 min, annealing temperature as listed in Table-2 for 1 min, 72°C for 1 min, and a final extension at 72°C for 10 min. The PCR products were resolved in 1.5% agarose gel containing 0.5 mg/ml ethidium bromide, and the size of amplicons was determined by comparison with a 100 bp DNA ladder (Genei^™^, Merck Bioscience, Bengaluru). The bands were visualized using a Gel documentation system (Bio-Rad, USA). The generated PCR products were sequenced (Bioserve Biotechnologies Limited, Hyderabad). The *V. parahaemolyticus* strains VP49 (which harbors the cas6-a genotype) and AQ4037 (reference strain) were used as positive and negative controls, respectively.

### *In silico* analysis

The *V. parahaemolyticus* cas6 sequences were downloaded from the National Centre for Biotechnology Information (https://www.ncbi.nlm.nih.gov/) database. The sequences obtained from our study were aligned with these sequences using the program MultAlin[22] and grouped based on similarity. The phylogenetic tree was generated using MEGA ver.5.0 [23].

## Results

### PCR for *cas6* gene

**Figure-1:**
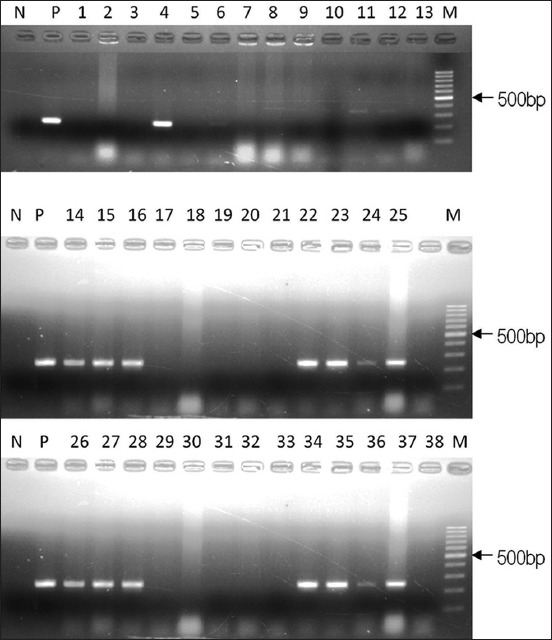
Polymerase chain reaction amplification of *cas6*-a subtype in *Vibrio parahaemolyticus*. N: Negative control; P: Positive control; M: 100 bp DNA marker; Lane 1-38: Environmental isolates of *V. parahaemolyticus*.

**Figure-2:**
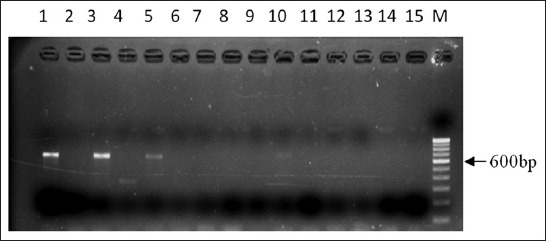
Polymerase chain reaction (PCR) amplification of *cas6*-d subtype in *Vibrio parahaemolyticus*. Only samples positive for PCR shown. Lanes 1-15: Environmental isolates of *V. parahaemolyticus*. M: 100bp DNA marker.

In this study, the primers designed were used to evaluate and detect the *cas6* gene subtypes in seafood and environmental isolates of *V. parahaemolyticus*. For initial identification of the type *cas6*-a, in *V. parahaemolyticus*, the PCR primers cas6-aF and cas6-aR (Table-2) were used, targeting an internal fragment of the gene. Among the 38 isolates studied, 12 (31.58%) amplified the expected product size of 245 bp (Figure-1) indicating the presence of *cas6*-a subtype in these strains. Similarly, three strains (VP1, VP5, and VPh3) were found to be positive for the type *cas6*-d variant and amplified a product size of 600 bp (Figure-2). However, *cas6*-b and *cas6*-c were not detectable in our strains. The remaining strains showed no amplification with any of the primers and hence considered negative for the presence of *cas6* gene. The PCR products of six isolates (Vp6, Vp8, Vp9, Vp10, Vp14, and Vp17) positive for the *cas6*-a gene variant were sequenced using the primers IF-a-F (forward) and IF-a-R (reverse) to generate a sequence of 597bp (Table-2). Two (Vp1 and Vp5) of the three strains positive for type *cas6*-d were also sequenced.

### *In silico* analysis of *V. parahaemolyticus cas6* sequences

**Figure-3:**
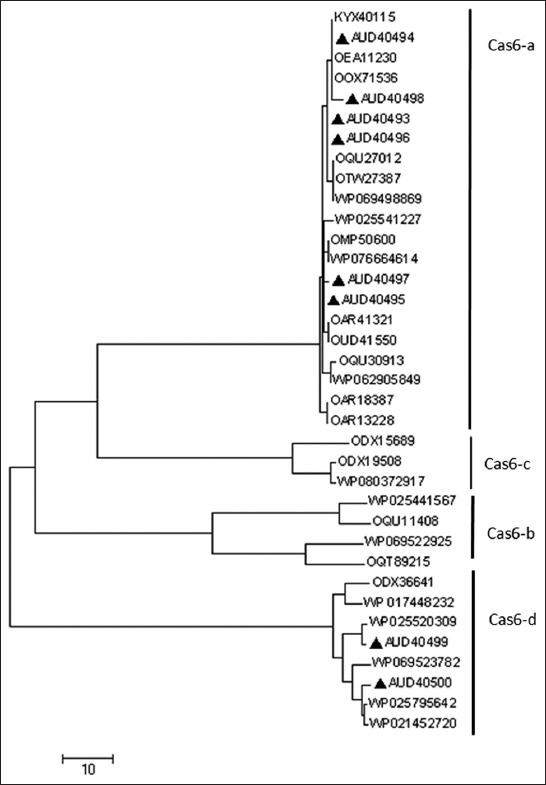
Phylogenetic tree of representative *cas6* protein sequences. Represents Cas6-a (AUD40493, AUD40494, AUD40495, AUD40496, AUD40497, and AUD40498) and Cas6-d (AUD40499 and AUD40500) subtypes identified in this study.

**Figure-4:**
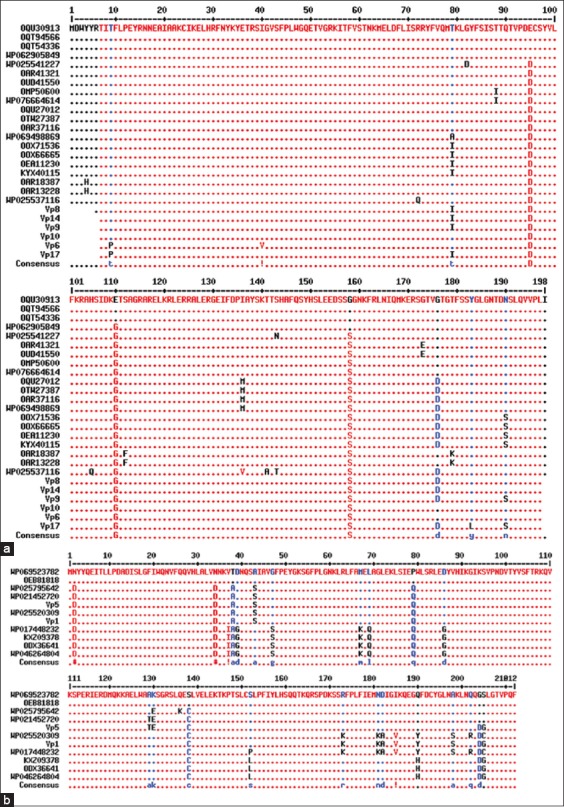
(a) Alignment of Cas6-a protein sequences of *Vibrio parahaemolyticus*. Vp6, Vp8-10, Vp14, and Vp17 represent environmental strains from this study. Amino acid variations among sequences are shown. (b) Alignment of Cas6-d protein sequences of *V. parahaemolyticus*. Vp1 and Vp5 represent environmental strains from this study. Amino acid variations among sequences are shown.

The phylogenetic tree generated based on Cas6 amino acid sequences was seen to subgroup into four major clusters (*cas6*-a-*cas6*-d, Figure-3). Pairwise alignment of representative Cas6 protein sequences from the different groups showed the sequences to be diverse with a homology of 21-47% between sequences. It was also seen that there exist residue differences within sequences within a given cluster (Figures-4a and b). Sequence analysis of the protein identified in this study showed proteins with accession IDs AUD40493, AUD40494, AUD40495, AUD40496, AUD 40497, and AUD40498 to be grouped along with the cas6-a and AUD40499 and AUD40500 grouped with Cas6-d proteins.

## Discussion

*V. parahaemolyticus* is known to harbor the type IF CRISPR-cas system [7]. *V. parahaemolyticus* subtype IF is associated with CRISPR-associated genes *cas1*, *cas3*, *cas8*, *cas5*, *cas7*, and *cas6*. However, a search for *cas* genes in this study showed that the majority (>90%) of *V. parahaemolyticus* strains contain a minimalistic type IF system containing core genes *cas5*, *cas7*, and *cas6*. Among these, the peripheral Cas6 domain belongs to the RAMP family of RNases functioning as an endonuclease that interacts with CRISPR RNAs to generate crRNAs [24,25]. Cas5 and Cas7 also belong to the RAMP superfamily and are implicated in interference and stabilization stages of crRNA generation [26]. Bioinformatics-based analysis of the *V. parahaemolyticus cas6* sequence showed the existence of four different sequence subtypes (*cas6*-a-*cas6*-d), with a majority of strains analyzed seen associated with *cas6*-a subtype [20]. The subtypes cas6-b and cas6-c were not detected in the strains studied which probably is due to the under-representation of environmental strains taken for analysis. A BLAST analysis for the *V. parahaemolyticus* *cas6*-b, *cas6*-c, and *cas6*-d subtypes showed homologous *cas6* to be present in *Vibrio anguillarum*, *Vibrio cholerae*, *Vibrio fluvialis*, *Vibrio vulnificus*, and several other *Vibrio* spp. There is much evidence that horizontal transfer of CRISPR and *cas* genes can occasionally occur between different strains, species, and even distant genera [26], which probably explains the diversity seen for *cas6* gene in *V. parahaemolyticus*. Cas6 belonging to the CRISPR-cas type IF system functions as an endonuclease that cleaves CRISPR RNAs to generate pre-crRNA [16]. Recent studies have shown that *cas6* (subtype IF) in *Pseudomonas* spp. recognizes its pre-crRNA substrate with high affinity, recognition of which is mediated by sequence and structure-specific interactions [17,27]. *V. parahaemolyticus* also contains the type IF system, but studies on its Cas6-mediated cleavage and the process to generate a pre-crRNA are lacking. Therefore, based on the diversity observed for *cas6* gene sequences in this study, we probably presume that, in *V. parahaemolyticus*, the Cas6 endonuclease could be functioning in more ways than one in recognizing the primary transcript and in bringing about cleavage. Further, in this study, an alignment of subtypes of *cas6*-a and *cas6*-d sequences revealed substitutions in several amino acid loci in the Cas6 protein (Figure-4a and b). This could be of significance, as studies show that mutations in any of the catalytic residues reduce the endonuclease activity of Cas6 resulting in non-cleaved pre-crRNA [27,28]. Whether these substitutions have any influence on the endonuclease activity, bringing about altered antiviral immunity, therefore, remains to be seen.

The gene coding for *cas6* endonuclease plays a critical role in the CRISPR cas-based adaptive immunity [29]. Studying the prevalence of the genes encoding these systems in seafood and/or environmental isolates of *V. parahaemolyticus* is expected to open new avenues in understanding the dynamics of the CRISPR-based immunity in this pathogen. Our study has elucidated the diversity of *cas6* gene in CRISPR-cas operon harboring *V. parahaemolyticus*. Thus, the PCR primers designed in this study could help in identifying and distinguishing the presence of *cas6* endoribonuclease variants in this pathogenic bacterium.

In the CRISPR-mediated immunity system, the *cas* genes are functionally paired with CRISPR repeats [30]. Thus, the absence of *cas6* gene in several of our environmental isolates indicates the presence of a sub-population of *V. parahaemolyticus* that probably lacks the CRISPR-cas system. To ascertain this, our future work would focus on studying CRISPR loci and finding an association with their presence/absence to *cas* genes in *V. parahaemolyticus*. Representative *cas6* genes pertaining to types *cas6*-a and *cas6*-d have been sequenced and deposited in GenBank having accession numbers MG417090 (strain Vp8); MG417091 (StrainVp9); MG417092 (StrainVp10); MG417093 (StrainVp14); MG417094 (StrainVp6); MG417095 (StrainVp17); MG417096 (StrainVp1); and MG417097 (StrainVp5).

## Conclusion

In prokaryotes harboring the CRISPR-cas type I-F system, the Cas6-associated protein functions as an endoribonuclease bringing about cleavage of the CRISPR RNAs and generation of pre-crRNA [16]. In *V. parahemolyticus*, a previous study with *cas6* showed the gene sequences to be diverse and broadly classified into four genotypes [20]. The primers designed in this study could be used in the identification of the *cas6* genotypes in *V. parahaemolyticus*. Further, our studies with seafood and environmental samples show the cas6-a variant to be most prevalent. Altered Cas6 could probably impact endoribonuclease activity. Thus, the proper identification of *cas6* genotypes in strains of *V. parahaemolyticus* is needed, which could contribute in understanding further the impact of such altered genotypes on the CRISPR–Cas immune system of this pathogenic bacterium.

## Authors’ Contributions

MS and MNV designed the study. MS designed the primers and suggested necessary steps involved in the research throughout the study. PB carried out the experimental work. PB and MS drafted the manuscript. MNV corrected the manuscript. All authors read and approved the final manuscript.
